# High Field MRI in Parotid Gland Tumors: A Diagnostic Algorithm

**DOI:** 10.3390/cancers17010071

**Published:** 2024-12-29

**Authors:** Chiara Gaudino, Andrea Cassoni, Martina Lucia Pisciotti, Resi Pucci, Chiara Veneroso, Cira Rosaria Tiziana Di Gioia, Francesca De Felice, Patrizia Pantano, Valentino Valentini

**Affiliations:** 1 Department of Neuroradiology, Azienda Ospedaliero-Universitaria Policlinico Umberto I, Viale del Policlinico 155, 00161 Rome, Italy; 2 Department of Oral and Maxillo-Facial Sciences, Sapienza University of Rome, Via Caserta 6, 00161 Rome, Italy; chiara.veneroso@uniroma1.it (C.V.); valentino.valentini@uniroma1.it (V.V.); 3 Department of Maxillo-Facial Surgery, Azienda Ospedaliero-Universitaria Policlinico Umberto I, Viale del Policlinico 155, 00161 Rome, Italy; 4 Department of Radiological, Oncological and Pathological Sciences, Sapienza University of Rome, Viale Regina Elena 324, 00180 Rome, Italy; martinalucia.pisciotti@uniroma1.it (M.L.P.); cira.digioia@uniroma1.it (C.R.T.D.G.); francesca.defelice@uniroma1.it (F.D.F.); 5 Department of Maxillo-Facial Surgery, Azienda Ospedaliera San Camillo Forlanini, Circonvallazione Gianicolense 87, 00152 Rome, Italy; rpucci@scamilloforlanini.rm.it; 6 Department of Human Neurosciences, Sapienza University of Rome, Viale dell’Univeristà 30, 00185 Rome, Italy; patrizia.pantano@uniroma1.it; 7 IRCCS Neuromed, Via Atinense 18, 86077 Pozzilli, Italy

**Keywords:** parotid gland, salivary gland tumors, magnetic resonance imaging, high field MRI

## Abstract

**Backgrounds:** Imaging of parotid tumors is crucial for surgery planning, but it cannot distinguish malignant from benign lesions with absolute reliability. The aim of the study was to establish a diagnostic MRI algorithm to differentiate parotid tumors. **Methods:** A retrospective study was conducted including all patients with parotid tumors, who underwent 3T-MRI and surgery. Morphological characteristics and normalized T2 and late postcontrast T1 signal intensities (SI) were assessed. “Ghosting sign” on late postcontrast T1 sequence was defined as indistinguishability of the tumor except for a thin peripheral enhancement. Patients were divided according to histology and imaging data were compared. A diagnostic MRI algorithm was established. **Results:** Thirty-six patients were included. The combination of normalized late T1 postcontrast SI, normalized T2 SI and “ghosting sign” allowed for the distinguishing of malignant from benign parotid tumors with high sensitivity (100%), specificity (93%), positive predictive value (80%), negative predictive value, (100%) and accuracy (94%). Moreover, pleomorphic adenomas often showed a homogeneous T2 signal and a complete capsule (*p* < 0.01), Warthin tumors protein-rich cysts and calcifications (*p* < 0.005 and *p* < 0.05), and malignant tumors an inhomogeneous contrast enhancement (*p* < 0.01). **Conclusions:** High field MRI represents a promising tool in parotid tumors, allowing for an accurate differentiation of malignant and benign lesions.

## Simple Summary

Preoperative evaluation of parotid tumors is crucial for treatment planning and relies on clinical examination, imaging techniques, and fine-needle aspiration cytology, although none of them can distinguish malignant from benign lesions with absolute reliability. The aim of our study was to evaluate MRI characteristics of the different parotid tumors and to establish a diagnostic MRI algorithm to use in the daily clinical practice to distinguish malignant from benign tumors. The results of this retrospective study showed that the combination of normalized late T1 postcontrast signal intensity, normalized T2 signal intensity and the “ghosting sign” allowed to distinguish malignant from benign parotid tumors with high sensitivity, specificity, positive predictive value, negative predictive value, and accuracy. High field MRI may, in the future, allow the limit of the use of preoperative, more invasive procedures, like fine-needle aspiration cytology, to selected and unclear cases.

## 1. Introduction

Salivary gland neoplasms represent approximately 3% of all head and neck tumors and can arise from both major and minor salivary glands [1]. Parotid gland tumors account for 85% of these neoplasms and are more frequently benign (80%), with pleomorphic adenoma (PA) the most common benign histotype, followed by Warthin tumors (WT) [2,3,4,5,6]. Aiming for complete tumor resection, surgery represents the first-line treatment, and it can vary from extracapsular dissection (ECD) to extended ECD, partial or total parotidectomy with or without neck dissection [7,8]. Preoperative evaluation is crucial for treatment planning as it varies according to the histotype, to tumor location, extension, and the presence of a solitary or multiple lesions. In patients with malignant parotid gland tumors, due to a higher risk of recurrence and worse prognosis, a total parotidectomy with free surgical margins is recommended. In higher grade malignancy or higher stage tumor, a neck dissection may be mandatory [9]. Moreover, for extensive malignant tumors, a neoadjuvant chemotherapy may be reasonable. Preoperative evaluation of patients with parotid tumors relies on clinical examination, ultrasound, computed tomography (CT), and magnetic resonance imaging (MRI), although none of them can distinguish malignant from benign lesions with absolute reliability [10,11,12,13,14,15,16,17,18,19,20,21]. Ultrasound-guided, fine-needle aspiration cytology (FNAC) is also recommended in the evaluation of patients with parotid tumors, but it is not mandatory. It is characterized by a high incidence of non-diagnostic results (5–21%) and variable low sensitivity and high specificity in the diagnosis of malignant tumors (64–90% and 86–100%, respectively), depending on tumor size, location, and pathologist’s experience [10,22,23,24].

Many research efforts have already focused on advanced MRI techniques in the differential diagnosis of parotid tumors with various combinations of dynamic contrast enhanced (DCE) imaging, diffusion weighted imaging (DWI), diffusion kurtosis imaging (DKI), intravoxel incoherent motion, and susceptibility weighted imaging (SWI), showing high variability in sensitivity, specificity, positive predictive, negative predictive, and accuracy values in distinguishing malignant from benign tumors (67–100%, 80–98%, 60–94%, 72–100%, 75–99%, respectively) [25,26,27,28,29,30,31,32,33,34,35,36,37,38,39]. Moreover, most of these techniques are difficult to apply in daily clinical practice due to an acquisition time increase and complex MRI data postprocessing.

Relative signal intensity (SI) measurements on conventional MRI sequences have the advantages of being more standardized and easier to reproduce across different scanners with varying field strengths, gradient configurations, or coil types. Moreover, they do not need complex postprocessing. To the best of our knowledge, only three studies have recently focused on signal intensity values on conventional MRI sequences in parotid tumors, with low comparability of results due to differences in MRI sequences analyzed and in tissues used as references to normalize the SI [27,40,41]. In two of these studies, the capability to distinguish between malignant and benign tumor was evaluated, showing promising results [27,40].

The aim of our study was to evaluate MRI characteristics of the different parotid tumors, focusing on relative SI, and to establish a diagnostic MRI algorithm to use in the daily clinical practice to distinguish malignant from benign tumors.

## 2. Materials and Methods

### 2.1. Patient Population and Study Design

A single-institution retrospective study was conducted. Inclusion criteria were (1) patients with newly diagnosed tumor of the parotid gland, who underwent (2) between April 2022 and August 2024 (3) surgery at the Department of Maxillo-Facial Surgery and (4) preoperative head and neck MRI at the Neuroradiology Department, both of University Hospital Policlinico Umberto I—Sapienza University of Rome, Italy.

Demographic and imaging data for all patients were retrospectively collected, as well as histopathological results on the surgical specimen. For those patients who underwent ultrasound guided FNAC before surgery, cytological results were also analyzed.

The study was conducted in accordance with the Declaration of Helsinki, and approved by the Institutional Review Board of University Hospital Policlinico Umberto I—Sapienza University of Rome (Prot. N. 0000007; 8 January 2024). Written informed consent was obtained from all participants.

### 2.2. Magnetic Resonance Imaging

All MRI examinations were performed on a 3.0 Tesla scanner (Magnetom Vida; Siemens Healthcare, Erlangen, Germany) using a BioMatrix 20-channel head and neck coil (Siemens Healthcare, Erlangen, Germany). Patients underwent MRI before FNAC. The standard MRI head and neck protocol for patients with parotid tumors at our institution includes T2 Short Tau Inversion Recovery (STIR), Turbo Spin Echo (TSE) T1 and T2 weighted, readout-segmented echo-planar diffusion weighted (rs-DWI), susceptibility weighted (SWI), 3D Double-Echo Steady-State with Water Excitation (DE3D-WE) sequences. Apparent diffusion coefficient (ADC) maps were generated automatically from DWI on the MRI acquisition workstation. During paramagnetic contrast medium iv injection (Dotarem^®^, Guerbet, Villepinte, France) a dynamic contrast enhanced (DCE) T1 Volumetric Interpolated Breath-hold (VIBE) perfusion sequence was acquired (51 repetitions of 6.6 s each; total acquisition time of 5:36 min). Contrast medium injection was administered using a power-injector (MEDRAD^®^ MRXperion, Bayer AG, Leverkusen, Germany) after the first four acquisitions (dose of 0.1 mmol/kg body weight, flow rate of 3.5 mL/second at a 20-G antecubital cannula). The contrast agent administration was followed by a power-injection of 20 mL saline at the same rate. After the perfusion weighted MRI, TSE T1 weighted fat saturated (FS) sequences were acquired on a coronal and axial plane. Due to acquisition time of the DCE and coronal T1 TSE FS sequences, the T1 TSE FS axial postcontrast sequence acquisition started at 9 min after contrast agent injection (late T1 TSE FS sequence). Sequences parameters are summarized in Table 1.

### 2.3. Post Processing of the Perfusion Data and Image Review

DCE MR images were analyzed using the Mean Curve function of the SYNGO.VIA View&Go VA.35 software (Siemens Healthcare, Erlangen, Germany). The T1 signal increase caused by the contrast agents was computed within a region-of-interest (ROI) obtaining signal intensity-time S(t)-curves. In the solid portion of the parotid lesions five or six ROIs (area ≥ 5 mm^2^) were positioned in consensus by two experienced radiologists (C.G., M.L.P.), with 10 and 4 years of experience, respectively, in head and neck imaging.

All MR images were displayed on a 30” flat panel In-Plane Switching (IPS) medical display (3280 × 2048 pixel) (Coronis Fusion 6 MP, Barco, Kortrijk, Belgium) using the Enterprise Imaging Platform (Agfa Healthcare, Mortsel, Belgium). The two radiologists in consensus, blinded to clinical and histopathological results, analyzed the images according to the following criteria:

#### 2.3.1. Qualitative Image Quality Analysis

The two observers were requested to describe the homogeneity of the tumor on T2-weighted and postcontrast T1-weighted sequences as homogeneous or inhomogeneous. They were also asked to identify the presence or absence of (1) T1 hyperintense cystic protein rich components in the lesions, (2) complete or incomplete capsule on the T2 weighted sequence as well as (3) hemorrhages and (4) calcifications on SWI. On late post-contrast axial T1 TSE FS sequence the presence of (5) the “ghosting sign”, defined as indistinguishability of the tumor from the surrounding normal parotid gland tissue except for a thin peripheral contrast enhancing ring, was also evaluated.

The parotid neoplasms were classified as superficial, deep or both according to the retromandibular vein [42,43].

#### 2.3.2. Quantitative Image Quality Analysis

The two radiologists were asked to measure the signal intensity (SI) of the solid portion of the tumor on TSE T2 and late axial postcontrast TSE T1 FS sequences by positioning, in consensus, a single ROI (area 10–15 mm^2^) per sequence. The ROI was localized in the solid portion of the lesion with the highest SI on late axial postcontrast T1 sequence and then in the same position on the T2 sequence. On each patient, the T2 and late T1 postcontrast SI values of the tumor were normalized according to the SI of the homolateral masseter muscle by positioning, by consensus, a single ROI (area 10–15 mm^2^) in the muscle belly and by calculating the lesion to muscle SI ratio. For all ROIs, the circle function of the Enterprise Imaging Platform was used.

Diffusivity values were measured on ADC maps by calculating the mean value of three circular ROIs (area 10–15 mm^2^) placed by consensus in the solid portion of the tumor.

The dynamic S(t)-curve were classified according to the time to peak and the washout ratio during acquisition time in type A (peak time > 120 s), type B (peak time ≤ 120 s and washout ratio ≥ 30%), type C (peak time ≤ 120 s and washout ratio < 30%), and type D (flat) curves, as described in the literature by Yabuuchi H. et al. [25].

### 2.4. Statistical Analysis

Data collection as well as descriptive statistics were carried out with Excel (Microsoft Office 2019) and SPSS software (IBM SPSS Statistics, version 26.0, IBM Corporation, Armonk, New York, NY, USA). Statistical differences were evaluated for continuous variables using one way ANOVA and student’s *t*-Test, for categorial data using the chi-square test. For all tests a level of *p* < 0.05 was considered as statistically significant.

Comparing the SI distribution in the different patient groups, suitable thresholds were defined to help to differentiate between the different histopathological diagnoses. Considering SI thresholds and the pathognomonic qualitative signs, sensitivity (SENS), specificity (SPEC), positive predictive value (PPV), negative predictive value (NPV), and accuracy of MRI in the diagnose of malignant tumor, pleomorphic adenoma, and Warthin tumor were calculated and compared, in our study population, with (1) the combination of DCE and DWI as proposed by Yabuuchi H. et al. [26] and (2) ultrasound guided FNAC values.

## 3. Results

We retrospectively analysed 38 consecutive patients with newly diagnosed parotid gland tumors, who underwent head and neck MRI and surgery at the University Hospital Policlinico Umberto I—Sapienza University of Rome, Italy. Two patients were excluded, one due to high melanin content in the lesion (pigmented epithelioid melanocytoma—PEM) and the other one because of complete cystic appearing lesion without solid component (lymphoepithelial cyst). The final study population included 36 patients (22 males and 14 females; mean age: 54.4 ± 17.4 years, range: 15–96 years).

According to the histopathological results, patients were divided in four groups: (1) pleomorphic adenoma (PA, n = 14, 39%); (2) Warthin tumor (WT, n = 10, 28%); (3) other benign tumors (OBT, n = 4, 11%) and (4) malignant tumor (MT, n = 8, 22%) (Table 2).

OBT included canalicular adenoma, myoepithelioma, oncocytoma, and reactive lymph node.

MT included low-grade mucoepidermoid Carcinoma (Ca), intraductal infiltrating Ca of salivary glands, Ca of the salivary ducts, acinar cell Ca, basal cell Ca of solid type, squamous cell Ca (two cases), and lymphoma.

Due to the small sample size and histopathological variability in OBT group, differences of qualitative and quantitative features were analyzed considering three groups: the two most frequent benign tumor groups (PA and WT) and malignant tumor patients. Three patients showed on MRI bilateral parotid tumors, but underwent surgery only for the more extensive lesion on one side. In two of three patients with bilateral lesions, the tumor was a Warthin tumor, and in one patient, an oncocytoma.

Thirty-one patients underwent ultrasound guided FNAC before surgery; in one patient, it was not performed due to parapharyngeal localization of the lesion and in four patients due to imminent surgery date. Four of 31 (13%) FNAC were non diagnostic. In 23 of 31 (74%) patients, the differentiation between malignant and benign lesion was correct; in four patients (13%), the result was wrong, with one false positive and three false negative results.

**Table 2:** Patients’ distribution according to histopathological results (Ca: Carcinoma).

	Number of Patients (n = 36)
**Benign lesions**	**28 (88%)**
Pleomorphic adenoma	14 (39%)
Warthin tumor	10 (28%)
Other benign tumors (OBT)	4 (11%)
Canalicular adenoma	1 (3%)
Myoepithelioma	1 (3%)
Oncocytoma	1 (3%)
Reactive lymph node	1 (3%)
**Malignant lesions**	**8 (22%)**
Low-grade mucoepidermoid Ca	1 (3%)
Intraductal infiltrating Ca	1 (3%)
Ca of the salivary ducts	1 (3%)
Acinar cell Ca	1 (3%)
Basal cell Ca of solid type	1 (3%)
Squamous cell Ca	2 (6%)
Lymphoma	1 (3%)

In bold the total number of benign and malignant lesions; Ca: Carcinoma

### 3.1. Qualitative Image Quality

Nineteen of thirty-six (53%) tumors affected both deep and superficial parotid lobe, 16 (44%) tumors were located only in the superficial portion of the gland and 1 (3%) tumor only in the deep one. Two superficial tumors were located at the lower pole and 5 in the anterior process of the parotid. The lesions showed a mean maximal diameter of 34.8 ± 18.6 mm (range: 11–82 mm) on T2 weighted images. Comparing PA, WT e MT groups the localization of the lesions was not significantly different in the three groups.

Morphological characteristics of the lesions in the three patient groups are summarized in Table 3.

T1 hyperintense protein-rich components were present in all Warthin tumors (100%) and only in 5 of 14 pleomorphic adenomas (36%) and in 3 of 8 malignant tumors (38%) (*p* < 0.005).

Pleomorphic adenomas showed more frequently a homogeneous structure on T2 (11 of 14 lesions—79%) compared to the other two groups (0 of 10 Warthin tumors and 2 of 8–25%—malignant tumors, *p* < 0.01).

A capsule on T2—weighted images was present in the most benign lesions (13 of 14 pleomorphic adenomas—93%—and 9 of 10 Warthin tumors—90%) and rarely in malignant tumors (3 of 8 lesions—38%) (*p* < 0.01). The capsule was complete in 10 of 14 (71%) pleomorphic adenomas, in none of the Warthin tumors and in 1 of 8 (13%) malignant tumors (*p* < 0.001).

Calcifications on SWI were present in all Warthin tumors and were less common in pleomorphic adenomas and malignant tumors (respectively 6 of 14–43%—pleomorphic adenomas and 6 of 8–75%-malignant tumors; *p* < 0.05). No significant differences were seen in the three groups concerning intralesional hemorrhages.

The “ghosting sign” on late T1 postcontrast axial sequence was present in all Warthin tumors and in none of the pleomorphic adenomas and of the malignant tumors (*p* < 0.001). Malignant tumors showed more frequently an inhomogeneous postcontrast enhancement compared to benign lesions (7 of 8–88%—malignant lesions versus 6 of 14–43%—pleomorphic adenomas and none of the Warthin tumors; *p* < 0.01).

**Figure 1:**
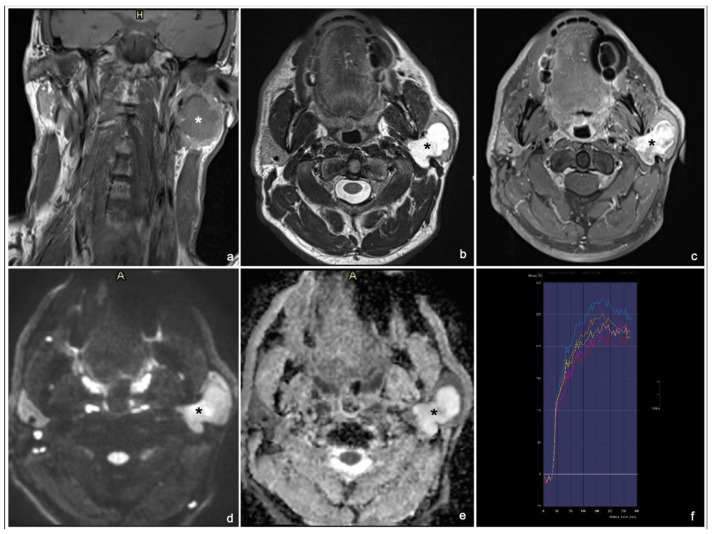
54 years old man with a pleomorphic adenoma of the left parotid gland: coronal T1 TSE (**a**), axial T2 TSE (**b**), late T1 TSE FS + Gd (**c**), DWI and ADC map (**d**,**e**) and DCE signal intensity-time S(t)-curve type A (**f**). * tumor. The lesion has an inhomogeneous, intense enhancement after contrast agent injection (normalized late T1 + Gd SI ≥ 2) (**c**) and a high signal on T2 (normalized T2 SI ≥ 3.5) (**b**).

**Figure 2:**
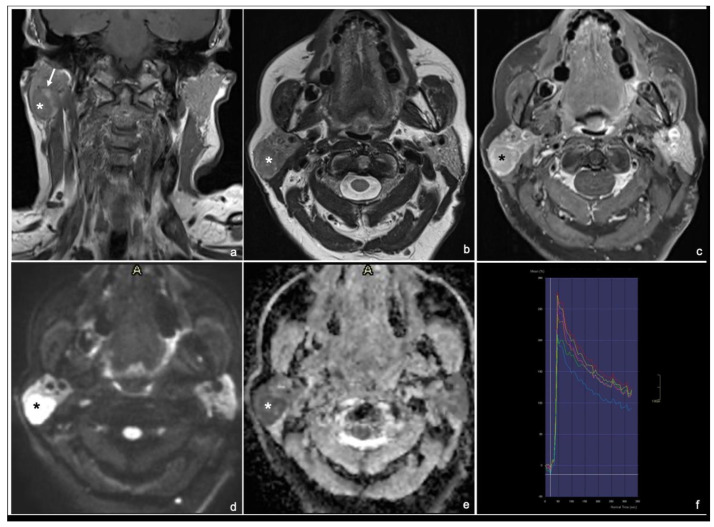
65 years old woman with Warthin tumor of the right parotid gland: coronal T1 TSE (**a**), axial T2 TSE (**b**), late T1 TSE FS + Gd (**c**), DWI and ADC map (**d**,**e**) and DCE signal intensity-time S(t)-curve type B (**f**). * tumor. The lesion is characterized by T1 hyperintense intratumoral cysts (arrow) (**a**) and low enhancement on late T1 post-contrast sequence (normalized late T1 + Gd SI < 2) with isointensity to the normal parotid tissue with a thin peripheral rim enhancement (“ghosting sign”) (**c**).

**Figure 3:**
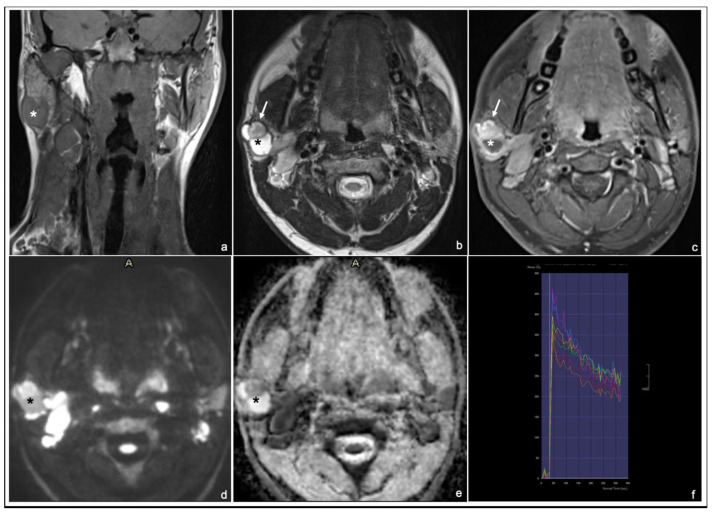
15 years old boy with low-grade mucoepidermoid Ca of the right parotid gland: coronal T1 TSE (**a**), axial T2 TSE (**b**), late T1 TSE FS + Gd (**c**), DWI and ADC map (**d**,**e**) and DCE signal intensity-time S(t)-curve type B (**f**). * tumor. The solid portion of the lesion (arrows) shows an intense enhancement on late T1 postcontrast sequence (normalized late T1 + Gd SI ≥ 2) (**c**) and appears hypointense in T2 (normalized T2 SI < 3.5) (**b**).

Typical MRI appearances of a pleomorphic adenoma, a Warthin tumor and a malignant tumor are shown in Figure 1, Figure 2 and Figure 3.

**Table 3:** Distribution of the morphological characteristics in the different parotid tumors.

	Pleomorphic Adenoma (n = 14)	Warthin Tumor (n = 10)	Malignant Tumor (n = 8)	*p* Value
Protein rich cysts	5 (36%)	**10 (100%)**	3 (38%)	<0.005
Homogeneity on T2w				
Homogeneous	**11 (79%)**	0 (0%)	2 (25%)	<0.01
Inhomogeneous	3 (21%)	10 (100%)	6 (75%)	
Capsule	**13 (93%)**	**9 (90%)**	3 (38%)	<0.01
Complete Capsule	**10 (71%)**	0 (0%)	1 (13%)	<0.001
Calcifications	6 (43%)	**10 (100%)**	6 (75%)	<0.05
Hemorrhages	8 (57%)	7 (70%)	5 (63%)	n.s.
Homogeneity on late T1 + Gd				
Homogeneous	8 (57%)	10 (100%)	1 (13%)	
Inhomogeneous	6 (43%)	0 (0%)	**7 (87%)**	<0.01
“Ghosting sign”	0 (0%)	**10 (100%)**	0 (0%)	<0.001

T2w: T2 weighted TSE sequence; T1 + Gd: T1 weighted postcontrast TSE sequence; Gd: Gadolinium containing contrast agent; n.s.: not significant. Significant values (*p* < 0.05) are in bold (chi-square test).

### 3.2. Quantitative Image Quality

**Figure 4:**
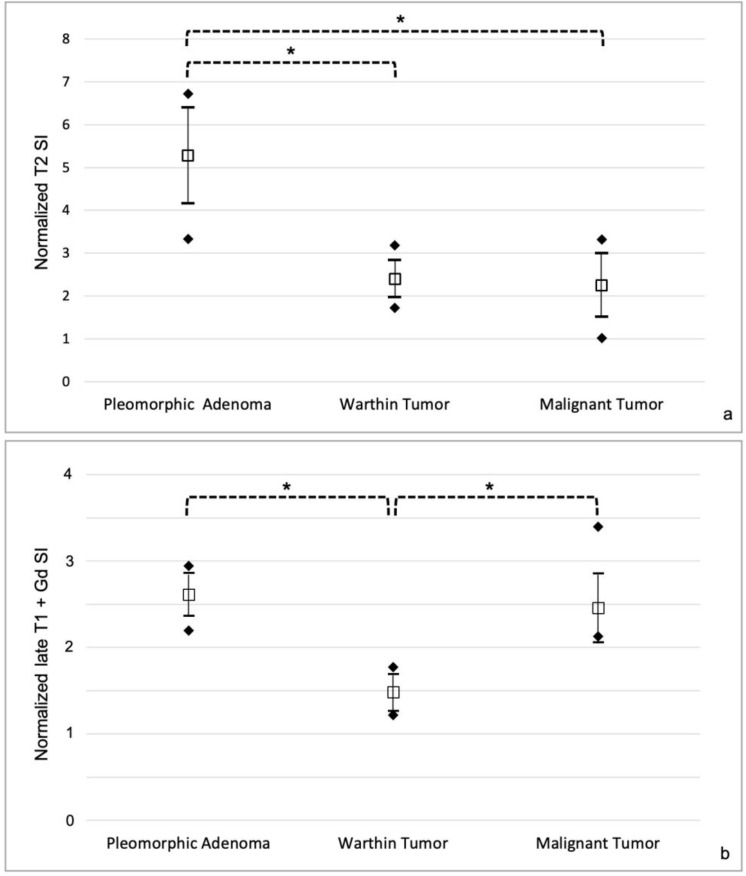
Normalized signal intensities in the three patient groups: Normalized T2 SI (**a**) and Normalized late T1 postcontrast SI (**b**). Data are shown as mean values ± standard deviation; ◆ minimum and maximum values in each group; * *p* < 0.001.

Normalized T2 SI was higher in pleomorphic adenomas than in Warthin tumors and malignant tumors (mean value 5.3 ± 1.1 compared to 2.4 ± 0.4 for WTs and 2.3 ± 0.7 for MTs; *p* < 0.001) (Figure 4a).

Normalized late T1 postcontrast SI was lower in Warthin tumors than pleomorphic adenomas and malignant tumors (mean value 1.5 ± 0.2 compared to 2.6 ± 0.2 for PAs and 2.5 ± 0.4 for MTs; *p* < 0.001) (Figure 4b).

Mean ADC values on DWI were higher in pleomorphic adenomas (2 ± 0.3 mm^2^ × 10^−3^/s versus 0.8 ± 0.1 mm^2^ × 10^−3^/s for Warthin tumors and 1.1 ± 0.4 mm^2^ × 10^−3^/s for malignant tumors; *p* < 0.001).

**Figure 5:**
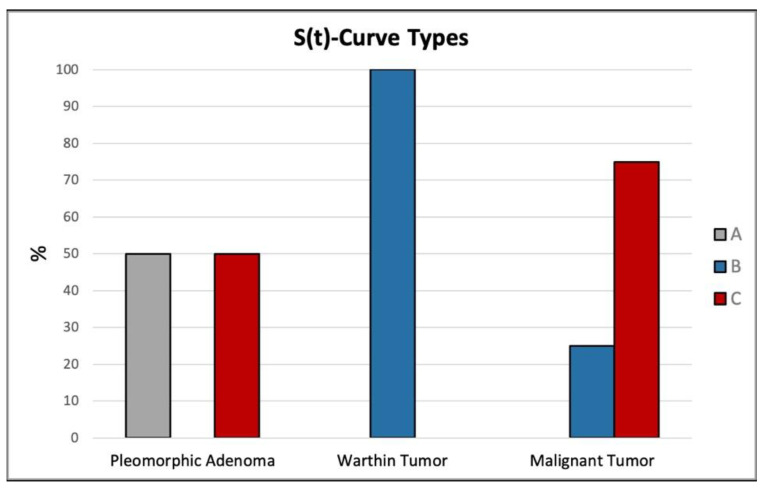
Distribution of the DCE signal intensity-time S(t)-curves types in the three patient groups, shown in percentages: A: type A, B: type B and C: type C curves.

The distribution of the different DCE signal intensity-time S(t)-curves types in the three groups is summarized in Figure 5. Type A curve was found in 8 of 36 (22%) patients (7 pleomorphic adenomas and 1 reactive lymph node), type B curve in 14 (39%) patients (10 Warthin tumors, 2 malignant tumors, 1 canalicular adenoma and 1 oncocytoma) and type C curve in 14 (39%) patients (7 pleomorphic adenomas, 6 malignant tumors and 1 myoepithelioma). The mean wash-out ratio of type B curve in the Warthin tumors was 64% ± 9 (range 47–79%) and in the malignant tumors 54% ± 6 (range 44–62%).

### 3.3. Diagnostic Algorithm

**Figure 6:**
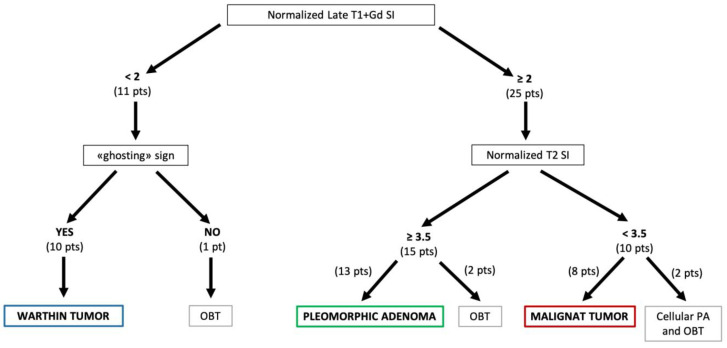
Diagnostic algorithm for head and neck MRI in patients with parotid tumors. (OBT: other benign tumors; pts/pt: patients/patient).

Comparing the distribution of normalized SI in the three patient groups (Figure 4) a threshold of <2 on late T1 postcontrast sequence and of ≥3.5 on T2 weighted sequence were defined for distinguishing respectively Warthin tumors and pleomorphic adenomas from malignant tumors. Considering these thresholds and the “ghosting sign” as a pathognomonic sign for Warthin tumors a diagnostic algorithm for the evaluation of head and neck MRI in patients with parotid tumors was established (Figure 6).

Using this diagnostic algorithm in our entire study population, MRI showed a sensitivity of 100% (8 patients of 8), a specificity of 93% (26 patients of 28), a positive predictive value (PPV) of 80% (8 patients of 10), a negative predictive value (NPV) of 100% (26 patients of 26) and an accuracy of 94% (34 patients of 36) in the differentiation of malignant from benign tumors compared to 76% (6 of 8), 86% (24 of 28), 60% (6 of 10), 92% (24 of 26) and 83% (30 of 36), respectively for the combination of DCE and DWI data and 63% (5 of 8), 78% (18 of 23), 83% (5 of 6), 86% (18 of 21) and 74% (23 of 31), respectively, for ultrasound guided FNAC (Table 4). The proposed MRI diagnostic algorithm allowed to identify correctly all malignant tumors; two benign lesions (a cellular pleomorphic adenoma and a myoepithelioma) were wrongly categorized as malignant tumors (false positive). On the other hand, using the combination of DCE and DWI data as proposed by Yabuuchi H. et al. [26] two malignant tumors were not correctly identified (false negative) and four benign lesions were wrongly seen as malignant tumors (false positive).

For the pleomorphic adenoma diagnosis, the MRI diagnostic algorithm showed a sensitivity of 93% (13 of 14), a specificity of 91% (20 of 22), a PPV of 87% (13 of 15), a NPV of 95% (20 of 21) and an accuracy of 92% (33 of 36); a canalicular adenoma and a reactive lymph node were wrongly categorized as pleomorphic adenoma (false positive). In the diagnose of Warthin tumors sensitivity, specificity, PPV, NPV and accuracy of the diagnostic algorithm were 100% (10 of 10, 26 of 26, 10 of 10, 26 of 26 and 36 of 36 respectively).

## 4. Discussion

The present study showed different morphological characteristics of parotid tumors on MRI. Benign tumors presented more often a peripheral capsule compared to malignant tumors. Pleomorphic adenomas had more frequently a homogeneous, high signal on T2 weighted images, often with a complete capsule. T1 hyperintense protein-rich cysts and calcifications were more frequent in Warthin tumors. Malignant tumors showed more frequently an inhomogeneous contrast enhancement than benign lesions due to necrosis. According to the results of the present study three features more clearly allowed a differential diagnose in parotid tumors: the normalized late T1 postcontrast SI with a threshold of 2, the normalized T2 SI with a threshold of 3.5 and the “ghosting sign” on late postcontrast T1 TSE FS sequences. They allowed to distinguish malignant tumors from Warthin tumors and pleomorphic adenomas with high sensitivity, specificity, PPV, NPV and accuracy.

Based on these features in our study population we could correctly identify all malignant tumors; the two false positive cases were wrongly categorized as malignant tumors due to high cellularity and consequential low signal intensity on T2.

The diagnostic MRI algorithm, established in the present study on the combination of these three features has showed in our patient population a higher diagnostic performance in the differentiation of malignant tumors from Warthin tumors and pleomorphic adenomas than DCE and DWI imaging data. DWI with the ADC maps and DCE with the different S(t) curve types may be of support in the diagnostic decision making, but do not allow, in our experience, to distinguish with absolute reliability malignant tumors from benign lesions. The diagnostic performance of the proposed MRI algorithm appeared higher also compared to ultrasound guided FNAC.

Concerning morphological MRI characteristics of the different parotid tumors, most of the results of the present study are in accordance with the literature with T1 hyperintense cysts more frequent in Warthin tumors [14,17,32,35], a higher T2 signal and the presence of a complete capsule in the most pleomorphic adenomas [12,13,14,19,27,32,35,38] and a more irregular contrast enhancement in malignant tumors [21,35]. Also, the ADC values on DWI and the distribution of the different S(t) curve types on DCE sequence in the three patient groups are in accordance with previous studies [18,25,26,38].

In our study population the parotid tumors did not differ in terms of intralesional hemorrhages on SWI, in contrast with the more extensive intratumoral susceptibility signal in malignant tumors reported by Xu Z. et al. [29], but the two studies are only partially comparable due to the simple dichotomy of the data in our study (presence or absence of hemorrhages) and the more complex grading system used by Xu Z. et al. (grade 0 to 3). To the best of our knowledges no previous studies have focused on differences in intralesional calcifications on SWI, while a higher frequency in Warthin tumors was observed in our study.

According to the present study the “ghosting sign” on the late postcontrast T1 FS axial sequence is likely to be pathognomonic for Warthin tumors. Many studies have described the low enhancement of Warthin tumors compared to pleomorphic adenomas and malignant tumors, but none of them used late postcontrast sequences (acquired at 9 min after contrast agent injection), like in our study [12,15,16,18,32,35]. The indistinguishability of the tumor from the surrounding normal parotid gland tissue except for a thin peripheral contrast enhancing ring (“ghosting sign”) on late postcontrast T1 FS images has, to the best of our knowledge, never been described so far and can be explained by the typical contrast agent dynamics in Warthin tumors with a rapid wash-in and a high washout ratio (peak time ≤ 120 s and washout ratio ≥ 30%, S(t) curve type B), as described in the literature [25,26]. The two patients with type B S(t) curve in the malignant tumor group showed a slight lower washout ratio than the patients with Warthin tumors. Due to the late acquisition of axial postcontrast T1 FS sequence in our study, the Warthin tumors have lost the majority of the contrast agent when this sequence was acquired.

The normalized T2 and late T1 FS signal intensity values in the present study are only partially comparable to previous studies in the literature, because of different sequence parameters used and different tissues chosen for normalization (spinal cord and normal parotid gland tissue) [40,41]. We preferred the masseter muscle as reference for normalization, because of the frequent artefacts at the level of the spinal cord and the higher variability of the parotid gland signal due to fatty degeneration and atrophy. Our normalized T2 signal intensity mean values for pleomorphic adenomas, Warthin tumors and malignant tumors are in accordance to the data published by Yabuuchi H et al. [27] (respectively 5.3 ± 1.1 for PA, 2.4 ± 0.4 for WT and 2.3 ± 0.7 for MT in our study compared to 4.0 ± 1.3 for PA, 2.3 ± 0.6 for WT and 2.4 ± 1.3 for MT in Yabuuchi H. et al.). In none of the previous studies post contrast T1 signal intensities were normalized with muscles.

The main limitation of the present study is its retrospective design with small sample (with few malignant tumor cases, of different histotypes), providing hypothesis generating rather than confirmatory results. Nonetheless, our study cohort is comprised of consecutive patients who were treated at a single institution with the same protocol management. The limited patient population did not allow to divide the PA in the different subtypes (classic PA, cellular PA and myxoid PA [41]) and to have an internal validation arm to evaluate the diagnostic algorithm. The proposed MRI diagnostic algorithm should be further confirmed with an external validation.

Recently more and more efforts are focusing on machine learning and MRI based radiomics models applied to head and neck tumors with promising results in the characterization of parotid gland tumors [44,45,46,47,48]. The MRI features pointed out in the proposed diagnostic algorithm could represent significant characteristics to take into account in radiomics models.

## 5. Conclusions

In conclusions, our study demonstrated the promising role of high field MRI in the preoperative evaluations of patients with parotid tumors, allowing an accurate differentiation of malignant and benign tumors based on three tumor features, which are normalized SI on late T1 postcontrast and on T2 images and “ghosting sign”. Further studies are needed to confirm these data on larger patient populations and the reproducibility of the proposed MRI diagnostic algorithm. It would be interesting to analyze if these results can be applied also to other salivary gland tumors. High field MRI may allow in future to limit the use of preoperative FNAC to selected and unclear cases.

## Figures and Tables

**Table 1:** Standard MRI protocol with sequence parameters for patients with parotid gland tumors (total acquisition time 34 min).

Sequence	TR (ms)	TE (ms)	Flip Angle (°)	Acquisitional Voxel Size	Averages	Acceleration	Acquisition (min)
T2 STIR cor	7770	38	144	1 × 1 × 3 mm	2	Grappa	3:45
T1 TSE cor	598	9.4	148	0.8 × 0.8 × 3 mm	2	Grappa	2:36
T2 TSE ax	5960	78	135	0.6 × 0.6 × 3 mm	2	Grappa	3:06
rs-DWI b50_800 ax	2880	66/103	180	0.7 × 0.7 × 4 mm	2/3	Grappa	5:56
SWI ax	28	20	15	0.6 × 0.6 × 1.6 mm	1	Grappa	2:15
DE3D-WE ax	14	5	30	0.3 × 0.3 × 0.5 mm	1	Grappa	4:59
DCE T1 VIBE + Gd ax	4.9	2.46	20	0.6 × 0.6 × 3 mm	1	Caipirinha	5:36
T1 TSE FS + Gd cor	777	9.4	160	0.8 × 0.8 × 3 mm	2	Grappa	3:22
T1 TSE FS + Gd ax	777	9.4	160	0.8 × 0.8 × 3 mm	2	Grappa	2:32

STIR: Short-Tau-Inversion Recovery sequence; T1 e T2 TSE: T1 weighted and T2 weighted Turbo Spin Echo sequence; rs-DWI: readout-segmented echo-planar diffusion weighted image; DCE T1 VIBE: dynamic contrast enhanced T1 weighted Volumetric Interpolated Breath-hold Examination; FS: Fat Saturation; Gd: Gadolinium containing contrast agent (Dotarem, Guerbet, Villepinte—France; 0.2 mL/kg); TR: Repetition Time; TE: Echo Time; cor: coronal; ax: axial.

**Table 4:** Diagnostic performance of the different methods in distinguishing malignant from benign tumors.

	Sensitivity (%)	Specificity (%)	PPV (%)	NPP (%)	Accuracy (%)
MRI Diagnostic Algorithm	100	93	80	100	94
DCE + DWI	76	86	60	92	83
FNAC	63	78	83	86	74

DCE: dynamic contrast enhanced imaging; DWI: diffusion weighted imaging; FNAC: fine-needle aspiration cytology; PPV: positive predictive value; NPP: negative predictive value.

## Data Availability

The data of this study are available from the corresponding author upon reasonable request.
